# Prevalence of Risk Factors of Non-communicable Diseases in a District of Gujarat, India

**DOI:** 10.3329/jhpn.v31i1.14752

**Published:** 2013-03

**Authors:** Aroor Bhagyalaxmi, Trivedi Atul, Jain Shikha

**Affiliations:** Department of Community Medicine, B.J. Medical College, Ahmedabad, Gujarat, India

**Keywords:** Cross-sectional study, Non-communicable diseases, Risk factors, WHO STEPS

## Abstract

The study attempted to identify the prevalence and distribution of risk factors of non-communicable diseases among urban and rural population in Gujarat, India. Using the WHO stepwise approach, a cross-sectional study was carried out among 1,805 urban and 1,684 rural people of 15-64 years age-group. Information on behavioural and physiological risk factors of non-communicable diseases was obtained through standardized protocol. High prevalence of smoking (22.8%) and the use of smokeless tobacco (43.4%) were observed among rural men compared to urban men (smoking-12.8% and smokeless tobacco consumption-23.1%). There was a significant difference in the average consumption of fruits and vegetables between urban (2.18±1.59 servings) and rural (1.78±1.48 servings) area. Prevalence of overweight and obesity was observed to be high among urban men and women in all age-groups compared to rural men and women. Prevalence of behavioural risk factors, overweight, and obesity increased with age in both the areas. Twenty-nine percent of the urban residents and 15.4% of the rural residents were found to have raised blood pressure, and the difference was found to be statistically significant (p<0.01). For both men and women, the prevalence of overweight and obesity, hypertension, and lack of physical activities were significantly higher in the urban population while smoking, smokeless tobacco consumption, poor consumption of fruits and vegetables were more prevalent in the rural population. The results highlight the need for interventions and approaches for the prevention of risk factors of non-communicable diseases in rural and urban areas.

## INTRODUCTION

India is a diverse country, and many states in India are passing through an epidemiological health transition with high rates of urbanization. Urbanization has led to economic improvement, the consequences of which is increased food consumption, tobacco-use, and decreased physical activity. One of the effects of this economic transition is a shift in the disease spectrum from communicable to non-communicable diseases (NCDs) (1). NCDs, especially cardiovascular disease, diabetes mellitus, and stroke, have emerged as a major public-health problem in India. The morbidity and mortality in most productive phase of life is posing serious challenges to Indian society and economy (2).

The huge burden of cardiovascular diseases in the Indian Subcontinent is the consequence of the large population and the high prevalence of CVD risk factors (3). NCDs have common risk factors, such as tobacco-use, unhealthy diet, physical inactivity, and excess adiposity. Policies and programmes focusing on reducing the burden of these common risk factors are likely to make a substantial impact on mitigating the mortality and morbidity due to NCDs (4).

Establishment of surveillance systems for non-communicable diseases and their risk factors is essential for developing prevention strategies and monitoring the impact of control programmes (5,6)

The WHO's approach to Surveillance of NCD Risk Factors (STEPS) was developed by WHO as part of a global surveillance strategy in response to the growing need for country-level trends in non-communicable diseases. By using the same standardized questions and protocols, all countries can use STEPS information not only for monitoring within-country trends but also for making between-country comparisons. The approach encourages the collection of small amounts of useful data on a regular and continuing basis. It focuses on a minimum number of risk factors that predict the major non-communicable diseases. This information can, in turn, be used in planning for disease prevention through population-level risk factor reduction (7,8).

In January 2008, the Ministry of Health and Family Welfare launched the pilot phase of the National Programme for Prevention and Control of Diabetes, CVD, and Stroke in 10 districts of the country. Gandhinagar is one of the districts selected, and the programme, among other things, aims at providing health promotion, screening for NCDs, risk factor surveillance, and setting up specialty clinics. For NCDs, while information on disease burden is useful for advocacy, the distribution of risk factors among the population is the key information required for the planning of prevention and control programmes. The information on risk factors predicts the future burden of diseases and is also useful to measure the effectiveness of prevention programmes (9). With this aim, the Department of Community Medicine, B.J. Medical College, Ahmedabad, carried out the survey in both urban and rural areas of Gandhinagar district in Gujarat state of India.

## MATERIALS AND METHODS

### Study setting

Gujarat is a prosperous state located in the western region of India. Percapita income in the state was approximately 63,961 Indian Rupees (equivalent to US$ 1,200) for the year 2010-2011, which is the fifth highest in the country. Also in 2009-2010, the state registered a growth of 12.99% gross domestic product, which is more than 11.2% set by the planning commission under the 11^th^ five-year plan (2007-2012). Gandhinagar district of Gujarat state was selected for the implementation of National Programme for Prevention and Control of Diabetes, Cardiovascular Diseases, and Stroke.

Gandhinagar district is an administrative division and capital of the Gujarat state, India. It has an area of 649 km² and a population of 1,334,455, of which 35.02% were urban (2001 Census). The study was conducted in both urban and rural areas of Gandhinagar district.

### Study group

Individuals aged 15-64 years residing in urban and rural areas of Gandhinagar district comprised the study group.

### Sample-size and sampling technique

As per the STEPS approach, 250 individuals were selected in each 10-year age interval for both sexes between 15 and 64 years. Estimated sample-size was 2500. To study the distribution of risk factors in both the areas, sample-size was increased to 3,500; 1,750 individuals were to be studied from each urban and rural area of Gandhinagar district. However, the actual numbers of samples included in the analysis are mentioned in the Abstract, the Results section, and the tables.

Sampling was done by multistage sampling technique which included the following three levels:

#### 1. Selection of sampling unit in urban and rural areas

Initially, selection of sampling unit in urban and rural areas was done as per the probability proportional to size (PPS). Villages in rural area and census enumeration blocks in urban area were taken as a sampling unit.

In rural area, 30 villages were selected as per probability proportional to size (PPS). The list of villages, along with their population in Gandhinagar district, was obtained from district health office, out of which 30 villages were selected. In urban area, 30 census enumeration blocks were selected as per PPS. The list of enumeration blocks and their population was obtained from census office.

#### 2. Selection of households in sampling unit

In rural area, 60 households were selected from each sampling unit (each village). The list of all households in the selected villages was obtained from the registers of the respective health workers. From this list, 60 households were selected by simple random technique, using random table.

In urban area, maps of the selected 30 blocks were obtained from census office. The list of all households in the selected enumeration blocks were obtained by the rapid survey conducted by the field investigators. From this list, 60 households were selected by simple random technique, using random table.

#### 3. Selecting individuals from the households

Both in urban and rural areas, one individual was selected from each household by the Kish method (9) involving a technique that allows for the random selection of one individual from a household. All persons above 55 years of age in the household were included in the study to have sufficient sample-size in the age-group of 55-64 years.

### Data collection

*STEP 1 (Interview):* Study protocol was based on the WHO STEPS approach. Information on sociodemographic variables and behavioural risk factors, such as tobacco-use, alcohol-use, physical exercise, and diet, were obtained by using a proforma translated in local language.

*STEP 2 (Physical measurements):* Height, weight, waist-circumference, and blood pressure were measured. Physical measurement, such as height and weight, was recorded to calculate BMI (kg/m^2^).

Blood pressure was measured using OMRON digital equipment recommended by Indian Council of Medical Research (ICMR) (OMRON-HEM7111, OMRON Healthcare Co. Ltd. Uky-Ku, Kyoto, Japan). Two readings were taken at an interval of 5 minutes, and the average value of the measurements was used for the analysis. Biochemical estimation could not be done due to limited fund.

### Definitions of risk factor

Overweight was defined as BMI equal to or more than 25 kg/m^2^, and ‘obesity’ as BMI equal to or more than 30 kg/m^2^. Waist-circumference ≥94 cm in men and ≥80 cm in women was taken as cutoff point to define central obesity (10). Low consumption of fruits and vegetables at less than five servings per day (one cup of raw leafy vegetables or half cup of other vegetables (cooked) was considered one serving. One medium-sized piece of fruit or half cup of chopped fruit was measured as one serving), and low physical activity was defined as <150 minutes of moderate physical activity per week. Raised blood pressure (hypertension) was defined if systolic blood pressure was ≥140 mm of Hg and/or diastolic pressure ≥90 mm of Hg, or diagnosed cases taking antihypertensive drugs (11).

### Data analysis

Data were analyzed using Epi Info software (version 3.5.1). Prevalence of different risk factors in different age-groups and sexes were analyzed for both rural and urban areas. Mean values of BMI, waist-circumference, and blood pressure were determined.

We obtained the ethical clearance from the ethical committee of the B.J. Medical College, Ahmedabad, Gujarat, India. Written informed consent was taken from all respondents.

## RESULTS

In total, 1,805 and 1,684 subjects were studied in rural and urban area respectively. Sociodemographic characteristics of the subjects are presented in Table 1. Prevalence and mean level of risk factors in different age-groups and in both the sexes are displayed in Table 2 and 3.

**T1able 1.** Sociodemographic profile of the study population

Variable	Urban	Rural
	(n=1,805)	(n=1,684)
Mean age (years)	37.8 (±1.36)	37.5 (±1.36)
Age-group (years) (%)		
15–24	21.4	20.8
>24–34	22.3	26.0
>34–44	24.7	21.9
>44–54	15.7	16.6
>54–64	16.0	14.7
Sex (%)		
Male	49.8	52.7
Female	50.2	47.3
Education (%)		
Illiterate	12.7	31.1
Primary	13.9	28.0
Secondary and higher secondary	55.1	39.4
College	10.4	0.7
Professional	7.9	0.8
Occupation (%)		
Business	7.0	4.3
Farming	0.7	16.7
Labour/unskilled work	3.5	20.1
Jobs/skilled work	31.8	10.0
Household work	26.3	10.9
Study/unemployed/retired	30.0	30.5

Only four women in the survey gave history of smoking in both the areas. However, the use of smokeless tobacco was reported by 5.5% and 19.8% of the women in urban and rural area respectively. High prevalence of smoking and the use of smokeless tobacco was observed among rural men compared to urban men in all age-groups, except in 15-24 years age-group (which was 1.6% in urban and 0.5% in rural area) (Table 3). Mean age of initiation of smoking in urban and rural men was 22.24±7.2 and 21.1±7.4 years compared to 24.58±10.2 and 21.9±10.0 years for smokeless tobacco consumption respectively. This difference in the mean age of initiation of both smoking and smokeless tobacco consumption between rural and urban men was statistically significant.

**T1able 2.** Prevalence of risk factors of non-communicable diseases

Risk factor	Male			Female			Total		
	No.	%	CI	No.	%	CI	No.	%	CI
Urban									
Daily smoking*	115/898	12.8	(10.58-15.02)	4/907	0.4	(0.0-0.8)	119/1,805	6.6**	(5.4-7.8)
Daily use of smokeless tobacco*	207/898	23.1	(20.3-25.9)	50/907	5.5	(4.0-7.0)	257/1,805	14.2**	(12.6-15.9)
Low physical activity*	500/898	55.7	(54.0-57.4)	202/907	22.3	(20.9-23.7)	702/1,805	38.9**	(37.8-40.1)
Low fruits and vegetables consumption	727/794	91.6	(90.6-92.6)	754/804	93.8	(93.3-94.3)	1,481/1,598	92.7**	(92.0-93.4)
Overweight	277/898	30.8	(27.7-33.9)	269/907	29.7	(26.7-32.7)	546/1,805	30.2**	(28.0-32.4)
Raised BP*	314/898	35.0	(31.8-38.2)	212/907	23.4	(20.6-26.2)	526/1,805	29.1**	(27.0- 31.2)
Central obesity*	197/898	21.9	(20.5-23.3)	491/907	54.1	(52.4-55.8)	688/1,805	38.1**	(37.0-39.2)
Rural									
Daily smoking*	203/887	22.8	(20.0-25.6)	4/797	0.5	(0-1)	207/1,684	12.29	(10.7-13.9)
Daily use of smokeless tobacco*	385/887	43.4	(40.1-46.7)	158/797	19.8	(17.0-22.6)	543/1,684	32.2	(29.9-34.5)
Low physical activity	131/887	14.8	(13.6-16.0)	108/797	13.6	(12.4-14.8)	239/1,684	14.2	(13.3-15.1)
Low fruits and vegetables consumption	850/887	95.8	(95.1-96.5)	774/797	97.1	(96.5-97.7)	1,624/1,684	96.4	(96.0-96.9)
Overweight	93/887	10.5	(8.44-12.6)	107/797	13.4	(11.0-15.8)	200/1,684	11.9	(10.3-13.5)
Raised BP	143/887	16.1	(14.9-17.3)	116/797	14.6	(13.3-15.9)	259/1,684	15.4	(14.5-16.3)
Central obesity*	48/880	5.5	(4.7-6.3)	191/773	24.7	(23.1-26.3)	239/1,653	14.4	(13.5-15.3)

*p<0.01 for male-female comparison;

**p<0.01 for rural-urban comparison; BP=Blood pressure; CI=Confidence interval

Urban residents were less physically active compared to rural residents. There was a significant difference in the average consumption of fruits and vegetables between urban and rural area (2.18±1.59 servings and 1.78±1.48 servings among urban and rural population respectively). Average intake of fruits and vegetables was significantly high among men in both the areas.

Compared to rural men and women, high prevalence of overweight and obesity was observed among urban men and women in all age-groups (average BMI was high in each age-group). There was no significant (p>0.05) gender difference in the mean BMI in both the areas; 54.1% of the urban women were found to have central obesity, and women in both the areas had significantly higher prevalence of central obesity compared to men.

Raised blood pressure was found in 29.1% of the urban residents and 15.4% of the rural residents. Increase in mean systolic and diastolic blood pressures was observed with increase in age, except in the age-group of 55-64 years in rural men and women. Significantly (p<0.01) higher prevalence of raised blood pressure was observed among both urban men and women compared to rural men and women. Gender difference in the prevalence was found in urban but not in rural area.

**T1able 3.** Prevalence of risk factors of non-communicable diseases stratified by age and sex

Age (years)	Daily smoker (%)	Mean systolic blood pressure	Mean diastolic blood pressure	Raised blood pressure (%)	Mean body mass index	Overweight or obese (%)
Urban						
Male (n=898)	12.8	130.7±16.6	81.3±11.6	37.3	23.2±4.6	30.8
15–24	1.6	126.8	76.9	21.8	20.7	12.9
>24–34	4.2	129.1	80.5	30.8	23.5	31.5
>34–44	8.1	129.6	82.5	36.2	23.6	35.8
>44–54	10.6	133.2	83.8	49.6	23.7	33.8
>54–64	10.4	137.0	83.9	46.0	25.1	43.9
Female (n=907)	0.4	125.9±17.3	79.4±16.5	27.3	23.1±4.8	29.7
15–24	-	118.7	76.9	11.4	20.2	10.3
>24–34	0.4	121.8	77.5	16.1	22.4	23.2
>34–44	0.4	126.0	80.0	27.3	23.9	34.5
>44–54	0.5	131.7	82.4	37.0	24.1	39.1
>54–64	-	136.3	81.9	58.2	25.6	49.3
Rural						
Male (n=887)	23.3	125.3±27.4	79.0±16.7	17.9	20.6±3.6	10.8
15–24	0.5	121.3	75.0	8.6	18.9	4.8
>24–34	15.1	123.4	78.8	13.7	20.4	7.3
>34–44	27.8	124.0	79.5	18.5	21.4	11.6
>44–54	49.3	127.0	83.7	21.2	21.1	17.8
>54–64	35.8	135.5	79.5	36.6	21.1	17.5
Female (n=797)	0.5	123.5±15.6	77.7±10.2	19.9	20.8±4.2	14.1
15–24	-	117.3	74.0	2.3	19.0	4.9
>24–34	0.5	120.5	76.8	9.6	20.3	10.5
>34–44	0.6	120.8	77.7	18.5	21.4	17.8
>44–54	1.5	129.8	80.7	21.2	21.4	18.0
>54–64	-	132.3	80.2	36.6	22.2	21.1

## DISCUSSION

Few studies in India have reported the prevalence of risk factors of non-communicable diseases in urban and rural areas (12,13,14). High prevalence of smoking among rural men compared to urban men is comparable with the findings of a study in Delhi conducted by ICMR in collaboration with WHO (15). NFHS-3 also reported higher prevalence of smoking for rural compared to urban region (16). However, overall prevalence of smoking observed in the present study (urban-12.8% and rural-22.8%) is low compared to the findings of a study in Delhi (urban-25% and rural-47.9%) (12). The prevalence in rural area only is comparable with the findings (24.3%) of a study done by ICMR at Kerala (14). The prevalence is also lower (55-60%) than that in an Indonesian study (17) and Maldives study (40%) (18). Women in both the areas had negligible rate of smoking (0.4-0.5%), suggesting a better influence of social norms. On the other hand, smokeless tobacco consumption was reported by women in both the areas. Gujarat being the second highest state in tobacco production, higher prevalence of smokeless tobacco consumption has been observed among both rural men and women in all age-groups compared to urban region. As a result, oral and oropharyngeal cancers are the leading malignant diseases in the rural area of Gujarat state (19). On the contrary, Vivek Gupta *et al.* (15) reported higher prevalence of smokeless tobacco consumption in urban area compared to rural area. Overall, the rural area reported high prevalence of tobacco-use either in the form of smoking or the consumption of smokeless tobacco compared to urban area. Bela Shah and colleague (20) also reported similar findings. The proportion of tobacco-use increased consistently with the increase in age, the highest being in 45-54 years age-group in both the areas. In the present study, the prevalence of smoking among urban adolescents and young adults (15-24 years) is more compared to the rural counterparts, suggesting chances of increased prevalence of smoking in urban area also in near future. Some start using tobacco as early as 10-12 years of age (average age of initiation of tobacco was between 20 and 25 years with a standard deviation of 7-10 years in both the areas). India is a signatory to the Framework Convention on Tobacco Control (FCTC) since September 2003. “The cigarettes and other tobacco products (Prohibition of Advertisement and Regulation of Trade & Commerce, Production, Supply & Distribution) Act 2003 was applied to whole of India. Early initiation of the use of tobacco forebodes serious public-health consequences. Tobacco control act has mandated the ban on tobacco sales to minors or near educational institutions and smoking in public places, etc. However, poor implementation of the act and easy availability of *pan masala, guthka,* areca nut, and slaked lime resulted in high prevalence of the use of smokeless tobacco (15).

Low consumption of fruits and vegetables probably could be due to the lack of awareness, especially in rural area. WHO attributes approximately three million deaths a year from non-communicable diseases to inadequate consumption of fruits and vegetables. Consumption of adequate fruits and vegetables not only prevents nutrient deficiency disorders but also reduces the risk of cardiovascular diseases. A study estimated that increased consumption of fruits and vegetables is associated with a 16% lower risk of cardiovascular disease (3). Availability of good-quality seeds, encouraging the farmers to grow vegetables and fruits, and public-awareness campaign about their benefits are some of the important strategies that may help increase the consumption of fruits and vegetables.

Since majority of the rural people are engaged in agricultural and labour work, only 10-15% of them reported to have less physical activity. Low physical activity observed among urban men (55.7%) and women (22.3%) in the present study is consistent with other urban surveys (12,13,21). Sedentary lifestyle of urban men and women has resulted in the high prevalence of overweight and obesity, which is almost three times higher in urban compared to rural area. This higher prevalence was observed in all age-groups and in both the sexes. No gender difference was observed. Many cross-sectional surveys recorded high and equal prevalence of central obesity and overweight with both men and women in urban area (13,14,22,23). Higher prevalence of central obesity was observed among females in the current study. Distribution of fat is as important as the total amout of fat in the body. Marked adverse metabolic consequences are seen with central obesity as this is also an independent predictor of non-insulin-dependent diabetes mellitus, coronary heart disease, NIDDM, CHD, hypertension, breast cancer, and premature death. Evidence from various studies indicates that obesity is preventable, and that the prevention of weight gain is easier, less expensive, and more effective than treating obesity and its complications. Policies to influence diet and physical activity should be based on multidisciplinary principles, and programmes should be based on evidence. Intersectoral collaboration among the stakeholders should also be encouraged.

The prevalence of hypertension has been reported to range between 20-40% in urban adults and 12-17% among rural adults (24). The present study also found high prevalence of raised blood pressure in urban area compared to rural area. Rural-urban variations may be explained partly by differences in physical activity and dietary habits. A study reported that higher intake of sodium is one of the important contributing factors for high blood pressure in urban population. This could be due to the excessive intake of ready-to-eat foods which are usually rich in sodium (12). This has been observed that high blood pressure is a major health problem in the elderly population of both sexes in both the areas. Raised blood pressure among young adults is a matter of concern as 16% and 6% of the urban and rural young adults (15-24 years) were found to have raised blood pressure. Priority should be given to school-based interventions, such as educating and exposing children to healthy food habits, encouraging daily physical activities, restricting the availability of high fat, salt, and sugar products within the proximity of schools. Although hypertension is highly prevalent, it is inadequately detected and treated. It was observed in the study that blood pressure of 50% of the rural participants has never been measured in the past.

While the age-specific rates of death from chronic diseases are declining in many high-income countries, the burden of these epidemics is accelerating in low- and middle-income countries driven by both ageing of population and rapid social and environmental changes that are contributing to the increase in the prevalence of common risk factors. At least 80% of heart disease, stroke, and type 2 diabetes and 40% of cancer could be avoided through healthy diet, regular physical activity, and avoidance of tobacco-use (4). A comprehensive strategy must integrate actions to minimize exposure to risk factors at the population level and reduce risk in individuals at high risk to provide early, medium-term and long-term effects (25).

### Conclusions

The high prevalence of risk factors of cardiovascular diseases both in urban and rural areas calls for a sound public-health approach. Efforts should be made to establish surveillance mechanism at the community level to monitor, evaluate, and guide policies and programmes. Multiplicative nature of risk factors suggests community-based behavioural and lifestyle-related interventions. To reduce the modifiable risk factors, interventions, such as tobacco control, production and supply of healthy foods, regulation of unhealthy foods, and urban planning to promote physical activity, need to be implemented. The health system has to forge partnerships within the different health programmes and also with other departments dealing with food and agriculture, industry, labour, education, and urban planning. In the present study, risk factors, like smoking, use of smokeless tobacco, and poor consumption of fruits and vegetables, are the areas of concern in the rural area. In urban area, the lack of physical activity, overweight, obesity, and prevalence of hypertension are the risk factors which need to be addressed. Results of the study highlight the need for different interventions and approaches for the prevention of risk factors of non-communicable diseases in rural and urban areas.

## ACKNOWLEDGEMENTS

The authors would like to thank Commissioner, Health, Medical Services & Medical Education, for financial support and District Health Officials for supporting the data collection by providing field investigators.
